# Cartilage tissues regulate systemic aging *via* ectonucleotide pyrophosphatase/phosphodiesterase 1 in mice

**DOI:** 10.1016/j.jbc.2023.105512

**Published:** 2023-11-30

**Authors:** Takahiro Arima, Kazuki Sugimoto, Takuya Taniwaki, Kazuya Maeda, Yuto Shibata, Makoto Tateyama, Tatsuki Karasugi, Takuya Tokunaga, Takanao Sueyoshi, Satoshi Hisanaga, Tetsuro Masuda, Yusuke Uehara, Masaki Yugami, Kozo Matsushita, Ryuji Yonemitsu, Junki Kawakami, Naoto Yoshimura, Shuntaro Tanimura, Hajime Kato, Nobuaki Ito, Kenichi Inoue, Kana Bando, Takayuki Nakamura, Takeshi Miyamoto

**Affiliations:** 1 Department of Orthopedic Surgery, Faculty of Life Sciences, Kumamoto University, Kumamoto, Japan; 2 Division of Nephrology and Endocrinology, The University of Tokyo Hospital, Tokyo, Japan; 3 Laboratory for Animal Resources and Genetic Engineering, RIKEN Center for Biosystems Dynamics Research, Kobe, Hyogo, Japan

**Keywords:** aging, bioluminescence, bone, calcification, cartilage, osteoporosis, vitamin D, metabolism

## Abstract

Aging presents fundamental health concerns worldwide; however, mechanisms underlying how aging is regulated are not fully understood. Here, we show that cartilage regulates aging by controlling phosphate metabolism *via* ectonucleotide pyrophosphatase/phosphodiesterase 1 (Enpp1). We newly established an Enpp1 reporter mouse, in which an EGFP-luciferase sequence was knocked-in at the *Enpp1* gene start codon (Enpp1/EGFP-luciferase), enabling detection of Enpp1 expression in cartilage tissues of resultant mice. We then established a cartilage-specific Enpp1 conditional knockout mouse (Enpp1 cKO) by generating Enpp1 flox mice and crossing them with cartilage-specific type 2 collagen Cre mice. Relative to WT controls, Enpp1 cKO mice exhibited phenotypes resembling human aging, such as short life span, ectopic calcifications, and osteoporosis, as well as significantly lower serum pyrophosphate levels. We also observed significant weight loss and worsening of osteoporosis in Enpp1 cKO mice under phosphate overload conditions, similar to global Enpp1-deficient mice. Aging phenotypes seen in Enpp1 cKO mice under phosphate overload conditions were rescued by a low vitamin D diet, even under high phosphate conditions. These findings suggest overall that cartilage tissue plays an important role in regulating systemic aging *via* Enpp1.

Aging is a complex physiological process experienced universally, although its rate of progression varies; how that rate is controlled, however, has not been characterized. Phosphate homeostasis is tightly regulated, and controlling phosphate metabolism is believed crucial to the aging process (1, 2, 3). Loss of either Klotho or FGF23 disrupts phosphate metabolism and in mouse promotes phenotypes mimicking human aging, including short life span and arteriosclerosis (4, 5, 6). Furthermore, loss-of-function mutations in either *KLOTHO* or *FGF23* reportedly underlie tumoral calcinosis, a disease characterized in humans by ectopic vascular calcifications (7, 8). Thus, the FGF23-Klotho axis is required and conserved to regulate phosphate metabolism and control the rate of aging in humans and mice.

Ectonucleotide pyrophosphatase/phosphodiesterase 1 (Enpp1), a transmembrane protein, generates pyrophosphate (PPi), which inhibits hydroxyapatite crystal deposition and mineralization in tissues (9, 10). *ENPP1* mutations have been detected in patients with autosomal recessive hypophosphatemic rickets type 2 or generalized arterial calcification of infancy (10, 11, 12, 13). *ENPP1* mutation is also seen in patients with ossification of the posterior longitudinal ligament, a disease characterized by ectopic ossification in spinal ligaments (14, 15), suggesting that ENPP1 plays essential roles in regulating phosphate metabolism and antagonizing ectopic calcification in humans. Furthermore, loss-of-function mutations in *Enpp1*, namely those seen in *Enpp1*^*ttw/ttw*^ (G1813 T) (16, 17) or *Enpp1*^*asj/asj*^ (V246D) mice (18), have been reported. *Enpp1*^*ttw/ttw*^ mice exhibit ossification of the posterior longitudinal ligament–like ectopic spinal ligament ossification (16, 17), whereas, *Enpp1*^*asj/asj*^ mice reportedly exhibit generalized arterial calcification of infancy–like phenotypes (18). *Enpp1*^*ttw/ttw*^ mice also exhibit premature aging phenotypes under phosphate overload conditions (6, 19, 20), similar to Klotho-mutant mice. Enpp1 is expressed in bone, cartilage, fat, heart, and liver tissues (21, 22, 23, 24), but it remains unclear which tissue is predominant in regulating aging phenotypes through Enpp1.

Here, we established mice with EGFP-luciferase knock-in at the Enpp1 gene and observed specific Enpp1 expression in cartilage tissues. We then established Enpp1 flox mice and used them to successfully generate cartilage-specific Enpp1 conditional knockout mice (Col2 Cre/Enpp1 cKO). Those mice exhibited various aging-related phenotypes including osteoporosis and ectopic calcium deposition in tissues, such as kidney and spinal ligament under phosphate overload conditions. Thus, we conclude that Enpp1 activity in cartilage is required to regulate global phosphate metabolism, ectopic ossification, and aging.

## Results

### Enpp1 is robustly expressed in chondrocytes

**Figure 1:**
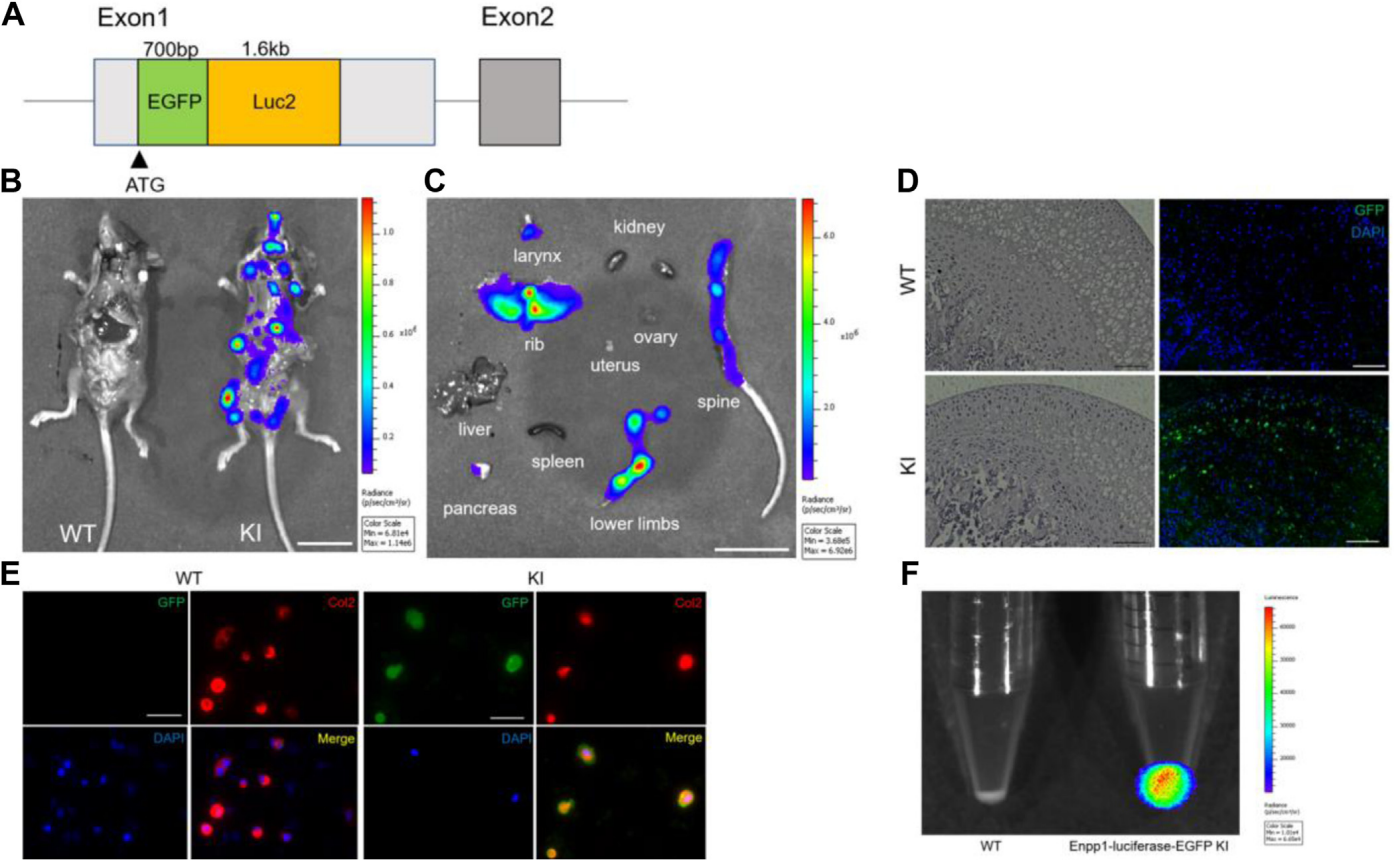
**Enpp1 is specifically expressed in chondrocytes.***A*, we generated reporter mice by knocking in a chimeric sequence, EGFP-firefly luciferase, at the start codon of exon 1 of mouse *Enpp1* (KI mouse). *B* and *C*, D-luciferin (0.15 mg/g body weight) was administered intraperitoneally to 4-week-old KI and wildtype (WT) mice, and 15 to 30 min later, *in vivo* imaging was performed (*B*). Bar, 20 mm. Luminescence signals were also monitored *ex vivo* in individual organs removed from KI mice (*C*). Bar, 20 mm. *D*, undecalcified frozen sections of femoral head were prepared in 4-week-old WT and KI mice, stained with HE or rabbit anti-EGFP antibody (diluted 1:1000) followed by Alexa488-conjugated goat anti-rabbit Ig’s antibody (diluted 1:400), and observed under a microscope (*left panels*) or a fluorescence microscope (*right panels*). Nuclei are stained with DAPI. Bar, 100 μm. *E*, primary chondrocytes were isolated from rib cartilage of 1-week-old WT or KI mice and stained with goat anti-EGFP antibody (diluted 1:250) and rabbit anti-Type2 collagen (diluted 1:200) followed by Alexa488-conjugated Donkey anti-Goat Ig’s (diluted 1:200) and Alexa594-conjugated Donkey anti-rabbit Ig’s antibodies (diluted 1:200). Nuclei were stained with DAPI. Cells were observed under a fluorescence microscope. Bar, 50 μm. *F*, pellet cultured cells shown in (*E*) were incubated 15 min with D-luciferin (0.5 mM), and chemiluminescence was analyzed. Enpp1, Ectonucleotide pyrophosphatase/phosphodiesterase 1; KI, knock-in.

To identify tissues expressing Enpp1 *in vivo*, we generated knock-in (KI) mice with a chimeric EGFP/firefly luciferase signal incorporated into Enpp1 exon1 (Fig. 1*A*). We then administered D-luciferin (0.15 mg/g) intraperitoneally to 4-week-old KI and wildtype (WT) mice and performed *in vivo* imaging analysis 15 to 30 min later. As expected, we detected no luminescence in WT mice, while KI mice displayed luminescence (Fig. 1*B*). For further analysis, we then dissected various organs from KI mice and observed strong luminescence in ribs, lower limbs, and spine, all of which contain cartilage regions, but almost no luminescence signals in kidney, liver, or spleen (Fig. 1*C*). To assess Enpp1 expression sites in greater detail, we performed immunofluorescence staining of EGFP on frozen sections of femoral heads from WT and KI mice. While no EGFP staining was evident in WT mice, articular cartilage and growth plate chondrocytes in KI mice were EGFP positive (Fig. 1*D*). We also isolated and cultured chondrocytes from rib cartilage of 1-week-old WT and KI mice and subjected them to immunofluorescent staining, followed by luminescence analysis 15 min after adding D-luciferin. Chondrocytes from KI mice were doubly positive for anti-type 2 collagen, a marker of chondrocytes, and anti-EGFP antibodies, whereas chondrocytes from WT mice were only positive for the anti-type 2 collagen antibody (Fig. 1*E*). Furthermore, KI cells exhibited luminescence, which was absent in WT cells (Fig. 1*F*), indicating specific expression of Enpp1 in chondrocytes.

### Global Enpp1-conditional knockout mice exhibit aging phenotypes

**Figure 2:**
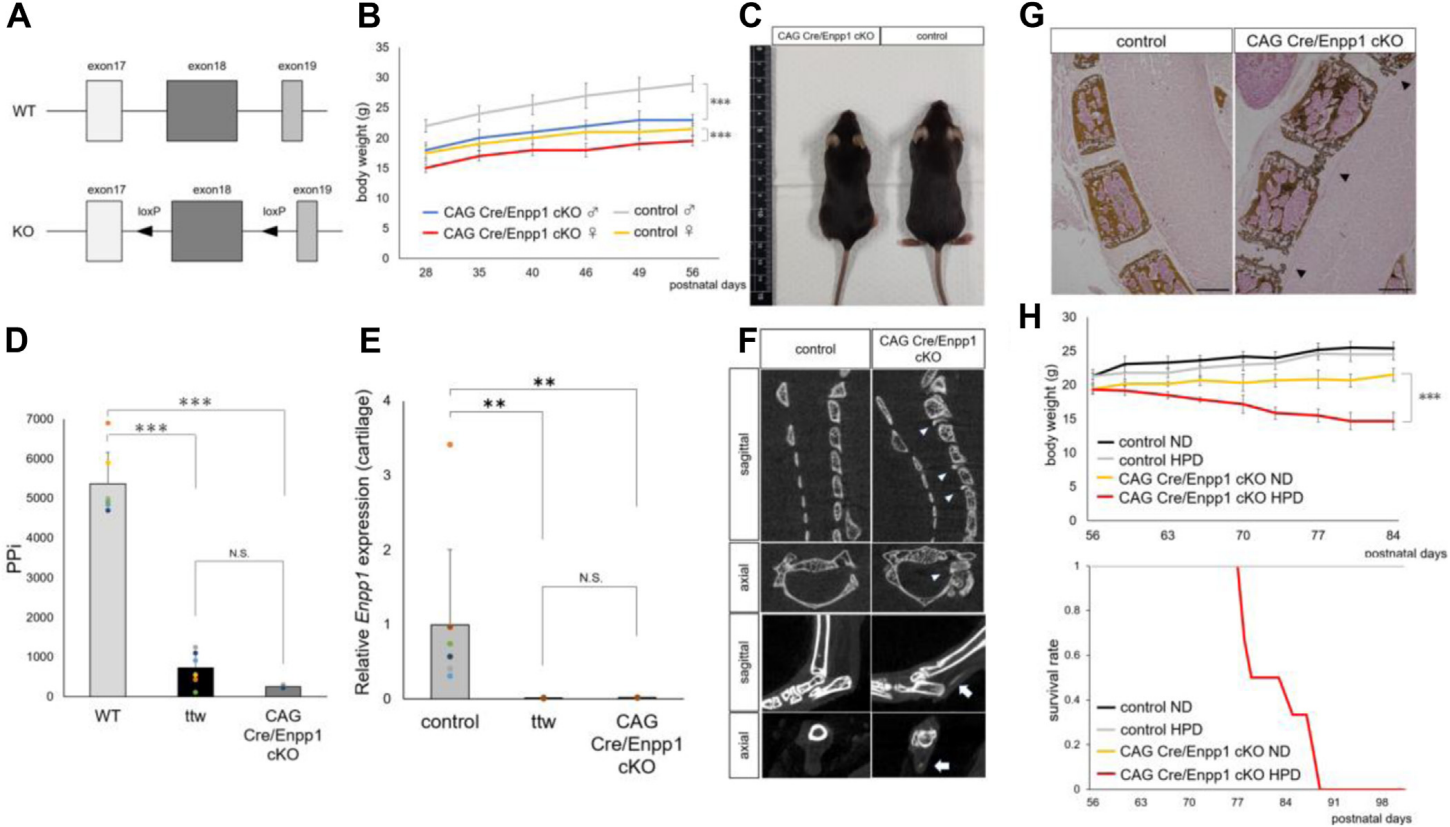
**CAG Cre/Enpp1 cKO mice exhibit phenotypessimilar to those seen in ttw mice.***A*, generation of Enpp1-flox mice. Mice with loxP sites flanking exon 18 were subsequently crossed with CAG Cre mice to yield CAG Cre; Enpp1flox/flox(CAG Cre/Enpp1 cKO) mice, in which Enpp1is globally deleted. *B*, changes in body weight in indicated cKO and control male and female mice over the period between 4 and 8 weeks of age. Data represent mean weight (g) of each time point ± SD (cKO, n = 10, control, n = 12, ∗∗∗*p* < 0.001).*C*, gross appearance of 8-week-old CAG Cre/Enpp1 cKO and control mice. *D*, RNA was extracted from head articular cartilage from 8-week-old *Enpp1flox/flox*(control), ttw, or CAG Cre/Enpp1 cKO mice, and *Enpp1*expression analyzed by real-time PCR. *E*, plasma was collected from 8-week-old mice in groups shown in (*D*), and plasma PPi was assessed using ATP sulfurylase. In (*D*and *E*) data represent mean *Enpp1*expression relative to *β-actin* (*D*) or plasma PPi ± SD (*E*), respectively(WT and ttw, CAG Cre/Enpp1 cKO; n = 6, ∗∗*p* < 0.01. ns, not significant). *F*, micro CT images of cervical spine (*upper*) and ankle joints (*lower*) of 8-week-old CAG Cre/Enpp1 cKO or *Enpp1flox/flox*(control) mice. *G*, undecalcified frozen sections of cervical spine from 8-week-old CAG Cre/Enpp1 cKO and *Enpp1flox/flox*(control) mice stained with von Kossa. *Arrowheads* in (*F* and *G*) indicate ectopic calcification. Bar, 100 μm. *H*, indicated groups of 8-week-old female were fed a normal (ND) or high-phosphate (HPD) diet, and changes in body weight (upper panel) and survival rate (lower panel) were monitored. Data represent mean body weight (g) at indicated time point ± SD (CAG Cre/Enpp1 cKO fed a ND or HPD; n = 6, control fed a ND or HPD; n = 10, ∗∗∗*p* < 0.001). CAG Cre/Enpp1 cKO mice, CAG Cre, Enpp1 flox/flox mice; Enpp1, Ectonucleotide pyrophosphatase/phosphodiesterase 1; KI, knock-in; PPi, pyrophosphate.

The above findings suggest that Enpp1 may regulate bone and phosphorus metabolism from chondrocytes. Thus, we generated Enpp1 flox/flox mice in which loxP sequences were incorporated at both ends of Enpp1 exon18 (Fig. 2*A*), since exon18 is mutated in ttw mice (17). We then crossed Enpp1 flox/flox mice with CAG Cre Tg mice to obtain CAG Cre, Enpp1 flox/flox mice (hereafter referred to as CAG Cre/Enpp1 cKO mice), which are globally Enpp1 deficient. CAG Cre/Enpp1 cKO mice exhibited tip-toe walking at 3 to 4 weeks of age (Fig. S1) and showed significantly reduced body weight compared to control mice (Cre-, Enpp1 flox/flox mice) (Fig. 2, *B* and *C*), both phenotypes seen in ttw mice. We then isolated total RNA from femoral head cartilage, bone, and small intestine tissues in CAG Cre/Enpp1 cKO mice and performed real-time PCR, which showed that Enpp1 expression levels were significantly decreased compared to those in control mice (Figs. 2*D* and S2). Moreover, Enpp1 expression levels in cartilage were comparable in CAG Cre/Enpp1 cKO and ttw mice (Fig. 2*D*). Since Enpp1 functions in PPi generation (9), we measured plasma PPi levels and found they were significantly decreased in CAG Cre/Enpp1 cKO compared to control mice (Fig. 2*E*). Micro CT imaging of cervical spine and ankle joints of 8-week-old CAG Cre/Enpp1 cKO mice revealed ectopic calcification around the intervertebral discs and Achilles tendon (Fig. 2*F*), both reportedly seen in ttw mice (25). We then performed von Kossa staining using frozen sagittal sections of cervical vertebrae from CAG Cre/Enpp1 cKO mice and observed ectopic calcification around intervertebral discs (Fig. 2*G*).

In ttw mice, high-phosphate diet (HPD) is known to cause early aging and weight loss phenotypes (19, 20). To determine whether CAG Cre/Enpp1 cKO mice exhibit similar phenotypes, we fed 8-week-old CAG Cre/Enpp1 cKO and control mice either a normal diet (ND) or a HPD. The CAG Cre/Enpp1 cKO HPD group exhibited significant weight loss compared to the three other groups, and all CAG Cre/Enpp1 cKO HPD mice died within 4 to 5 weeks (Fig. 2*H*). Overall, we conclude that Enpp1 flox/flox mice can successfully be made conditionally deficient in Enpp1 using the Cre-loxP system and that resulting CAG Cre/Enpp1 cKO mice exhibit phenotypes comparable to ttw mice.

### Cartilage-specific Enpp1 conditional knockout mice exhibit aging phenotypes

Type2 collagen is reportedly expressed in tissues such as cartilage, the nucleus pulposus of intervertebral discs and the vitreous body, but is primarily expressed in chondrocytes (26, 27). To assess effects of Enpp1 loss in chondrocytes, we crossed Enpp1 flox/flox mice with Type 2 collagen Cre Tg mice to eliminate Enpp1 specifically in chondrocytes (hereafter referred to as Col2 Cre/Enpp1 cKO mice). Indeed, we detected chondrocyte-specific EGFP expression in Col2 Cre/loxP-EGFP reporter mice (Fig. S3), which showed specific EGFP expression in type 2 collagen-expressing cells. After immunostaining for EGFP, sagittal frozen sections of cervical vertebrae from these mice exhibited specific EGFP staining in the nucleus pulposus in intervertebral discs and in growth plate chondrocytes in endplates (Fig. S3).

**Figure 3:**
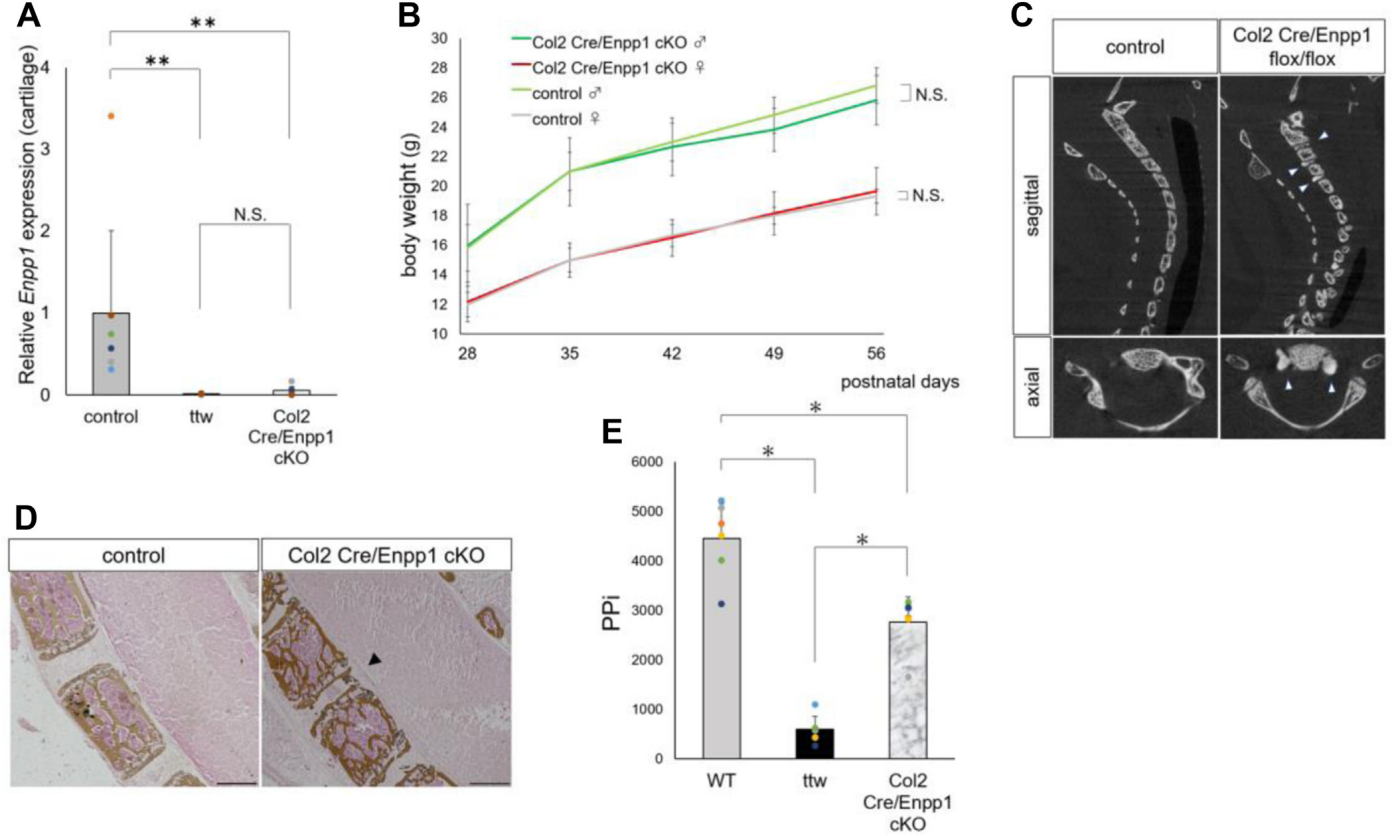
**Col2 Cre/Enpp1 flox mice exhibit phenotypes similar to those seen in ttw mice.** Col2 Cre and Enpp1-flox mice were crossed to yield Col2 Cre; *Enpp1flox/flox* (Col2 Cre/Enpp1 cKO) mice, in which Enpp1is specifically knocked out in chondrocytes. *A*, RNA was extracted from femoral head articular cartilage in indicated 8-week-old mice and *Enpp1*expression analyzed by real-time PCR. Data represent mean *Enpp1*expression relative to *β-actin* (*A*) (n = 7, ∗∗*p* < 0.01. ns, not significant). *B*–*D*, Col2 Cre/Enpp1 cKO or *Enpp1flox/flox*(control) male and female mice were fed a ND for 8 weeks. *B*, changes in body weight in indicated groups over a period when mice were 4 to 8 weeks of age. Data represent mean weight (g) of each time point ± SD (n = 6, ns, not significant). *C*, micro CT imaging of cervical spine of indicated 8-week-old mice. *D*, undecalcified frozen sections of cervical spine from indicated 8-week-old mice stained with von Kossa. *Arrowheads* in (*C* and *D*) indicate calcification. Bar, 100 μm. *E*, plasma was collected from 8-week-old wildtype, ttw, and Col2 Cre/Enpp1 cKO mice, and plasma PPi was assessed using ATP sulfurylase. Data represent mean plasma PPi ± SD (n = 6, ∗*p* < 0.05. ns, not significant). Col2 Cre/Enpp1cKO mice, type 2 collagen Cre; Enpp1 flox/flox mice; Enpp1, Ectonucleotide pyrophosphatase/phosphodiesterase 1; PPi, pyrophosphate.

We then performed real-time PCR from total RNA from femoral head cartilage of Col2 Cre/Enpp1cKO mice and observed significantly decreased Enpp1 expression levels relative to control (Enpp1 flox/flox) mice (Fig. 3*A*). No significant decrease in Enpp1 expression relative to controls was seen in other tissues from Col2 Cre/Enpp1cKO mice (Fig. S4). Although Col2 Cre/Enpp1 cKO and control mice showed comparable body weight (Fig. 3*B*) and Col2 Cre/Enpp1 cKO mice did not exhibit tip-toe walking at 8 weeks of age, micro CT imaging of the cervical spine revealed ectopic calcification around the intervertebral disc at the 8-weeks’ time point in Col2 Cre/Enpp1 cKO mice (Fig. 3*C*). Von Kossa staining of cervical spine confirmed ectopic calcification in the same area detected by micro CT in Col2 Cre/Enpp1 cKO mice (Fig. 3*D*). Plasma PPi levels were significantly reduced in 8-week-old Col2 Cre/Enpp1cKO relative to WT mice (Fig. 3*E*). These results indicate that Col2 Cre/Enpp1 cKO mice recapitulate phenotypes seen in ttw and CAG Cre/Enpp1 cKO mice, although phenotypes in Col2 Cre/Enpp1 cKO mice are milder.

### Col2 Cre/Enpp1 cKO mice exhibit systemic aging phenotypes

**Figure 4:**
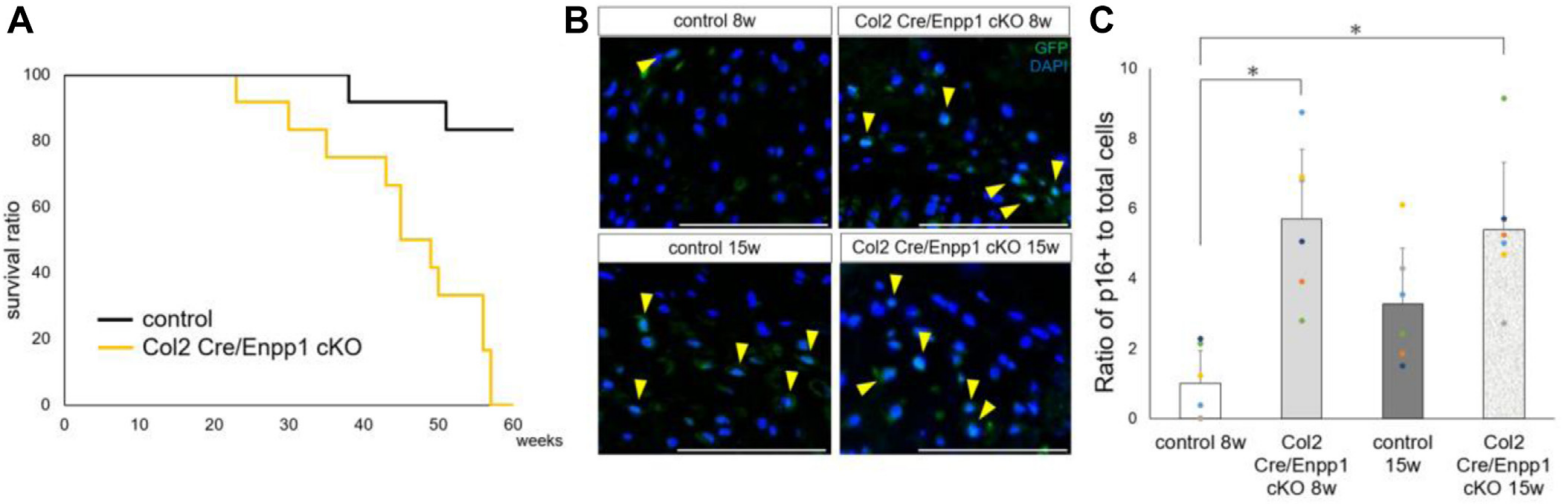
**Col2 Cre/Enpp1 cKO mice exhibit systemic aging phenotypes.***A*, weeks of survival were quantified using the Kaplan–Meier log rank survival test (each group; n = 12). *B*, undecalcified frozen sections of cervical spine were prepared from 8- to 15-week-old *Enpp1flox/flox*(control) or Col2 Cre/Enpp1 cKO mice, stained with rabbit anti-p16INK4a antibody followed by Alexa488-conjugated goat anti-rabbit Ig antibody, and observed by fluorescence microscopy. Nuclei were DAPI stained. *Arrowheads* in (*B*) indicate p16-positive cells. Bar, 25 μm. *C*, the number of p16-positive cells relative to the total number of DAPI-stained nuclei in the intervertebral disc was scored. All values represent means ±SD (n = 6, ∗*p* < 0.05). Col2 Cre/Enpp1cKO mice, type 2 collagen Cre; Enpp1 flox/flox mice; Enpp1, Ectonucleotide pyrophosphatase/phosphodiesterase 1.

Since Col2 Cre/Enpp1 cKO mice show phenotypes similar to those seen in ttw mice (specifically, phenotypes resembling human aging (20)), we performed longitudinal studies of aging in Col2 Cre/Enpp1 cKO mice (Fig. 4). Relative to control mice, Enpp1 cKO mice had a significantly shorter life span (Fig. 4*A*). We also analyzed the percentage of p16-positive cells in intervertebral disc tissues in Col2 Cre/Enpp1 cKO and control mice, since p16 is a known marker of cell senescence (28). At 8 weeks of age, the percentage of p16-positive cells was significantly higher in Enpp1 cKO than in control mice. However, at 15 weeks of age, those percentages remained higher in Enpp1 cKO compared to control mice, but differences were not significant (Fig. 4, *B* and *C*). The frequency of mesenchymal stem/progenitor cells, defined as CD45-negative/CD31-negative/PDGF receptor alpha-positive cells, in bone marrow was comparable between genotypes at 8 weeks of age (Fig. S5) and also comparable to the frequency seen in control mice at 15 weeks; however, that frequency was decreased in Enpp1 cKO relative to control mice at 15 weeks, although the difference was not significant (Fig. S5).

### Phenotypes seen in Col2 Cre/Enpp1 cKO mice are accelerated by high phosphate conditions

**Figure 5:**
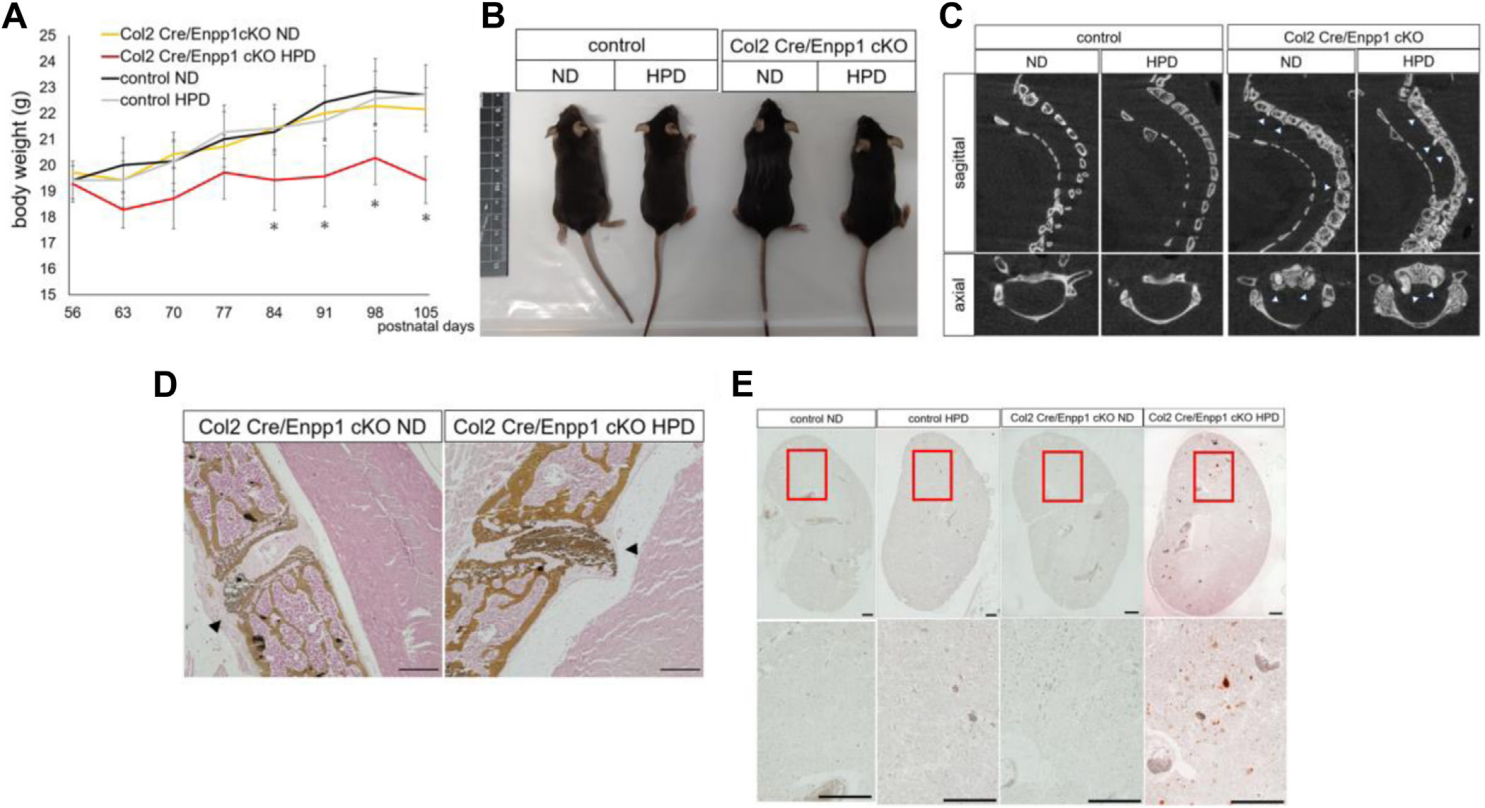
**A high-phosphate diet promotes weight loss in Col2 Cre/Enpp1 cKO mice.** Eight-week-old Enpp1flox/flox (control) and Col2 Cre/Enpp1 cKO mice were fed a normal diet (ND) or high-phosphate diet (HPD) for 7 weeks (*A*–*E*). *A*, changes in body weight in indicated groups over a period when mice were 8 to 15 weeks of age. Data represent mean weight (g) of each time point ± SD (n = 7, ∗*p* < 0.05). *B*, gross appearance of 15-week-old mice in indicated groups. *C*, micro CT imaging of cervical spine of 15-week-old mice from indicated groups. *D*, undecalcified frozen sections of cervical spine were prepared in indicated 15-week-old mice and stained with von Kossa. In (*C* and *D*) *arrowheads* indicate ectopic calcification. Bar, 100 μm. *E*, undecalcified frozen sections of kidney were prepared in indicated 15-week-old mice and stained with Alizarin Red. Regions boxed in *upper panels* are shown at high magnification in adjacent *lower panels*. Bar, 500 μm. Col2 Cre/Enpp1cKO mice, type 2 collagen Cre; Enpp1 flox/flox mice; Enpp1, Ectonucleotide pyrophosphatase/phosphodiesterase 1.

Next, we fed 8-week-old Col2 Cre/Enpp1 cKO and control (Enpp1 flox/flox mice) mice ND and HPD diets, since ttw mice fed a HPD show weight loss and accelerated ectopic calcification in tissues, such as spine and kidney (20). Col2 Cre/Enpp1cKO mice fed a HPD showed significant weight loss compared to Col2 Cre/Enpp1 cKO mice fed a ND or controls fed a HPD for the same time period (Fig. 5, *A* and *B*). Micro CT imaging performed in 15-week-old mice after 7 weeks on the ND or HPD revealed ectopic ossification around the intervertebral disc in Col2 Cre/Enpp1cKO mice fed a ND, which was enhanced in Col2 Cre/Enpp1 cKO mice fed a HPD (Fig. 5*C*). Von Kossa staining of cervical spine confirmed worsened ectopic calcification, as detected by micro CT in Col2 Cre/Enpp1 cKO mice fed the HPD (Fig. 5*D*). We also detected accelerated ectopic calcification in kidneys of 15-week-old Col2 Cre/Enpp1 cKO mice fed the HPD (Fig. 5*E*). No obvious ectopic calcification in kidney was observed in either control group or in Col2 Cre/Enpp1 cKO mice fed a ND (Fig. 5*E*). We conclude that phosphate supplementation accelerates aging phenotypes in Col2 Cre/Enpp1 cKO mice, as seen in ttw mice. ttw mice fed a HPD showed reduced expression of the *Klotho* gene, which encodes a coreceptor of fibroblast growth factor receptor, but those mice exhibited relatively higher *Cyp27b1* transcript levels in kidney (20). Indeed, 15-week-old Col2 Cre/Enpp1 cKO mice fed a HPD for 7 weeks exhibited significantly reduced *Klotho* transcript levels, as seen in kidney of 10-week-old ttw mice (Fig. S6). Moreover, *Cyp27b1* expression in kidney was comparable in 15-week-old control mice fed either a ND or a HPD for 7 weeks, whereas it was significantly upregulated in kidney of 15-week-old Col2 Cre/Enpp1 cKO mice fed a HPD for 7 weeks, as seen in similarly treated *ttw* mice (Fig. S7).

### Phenotypes seen in Col2 Cre/Enpp1 cKO mice are rescued by a low vitamin D diet

**Figure 6:**
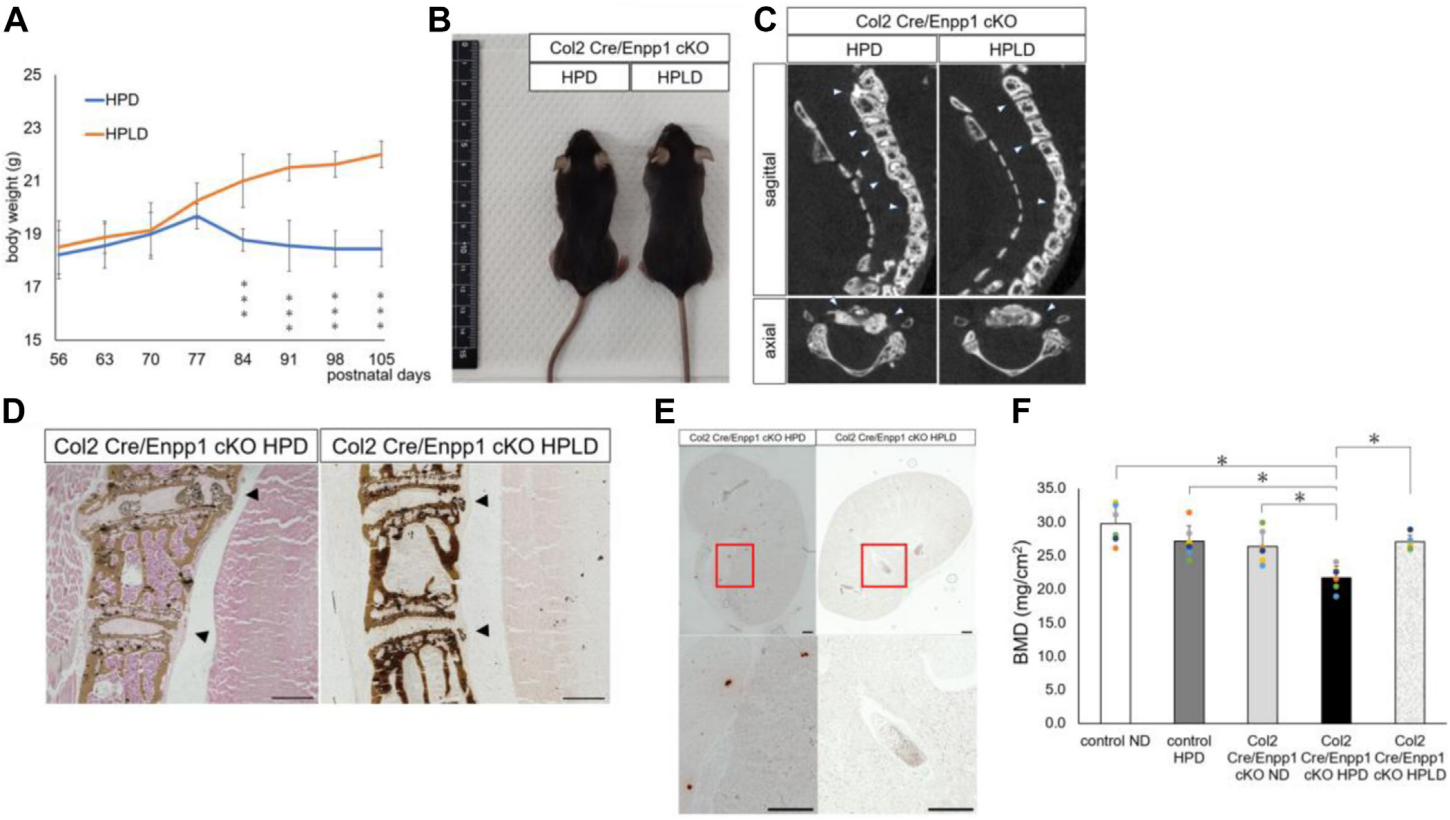
**A low vitamin D diet antagonizes aging phenotypes seen in Col2 cKO mice under phosphate overload.** Eight-week-old Col2 Cre/Enpp1 cKO mice were fed a HPD or a high phosphate/low vitamin D diet (HPLD) for 7 weeks (*A*–*F*). *A*, changes of body weight in indicated groups over a period when mice were 8 to 15 weeks of age. Data represent mean weight (g) of each time point ± SD (n = 8, ∗∗∗*p* < 0.001). *B*, gross appearance of 15-week-old indicated mice. *C*, micro CT imaging of cervical spine of indicated 15-week-old mice. *D*, undecalcified frozen sections of cervical spine from 15-week-old indicated mice stained with von Kossa. In (*C* and *D*) *arrowheads* indicate calcification. Bar, 100 μm. *E*, undecalcified frozen sections of kidney from 15-week-old indicated mice stained with Alizarin *Red*. Regions boxed in *upper panels* are shown at high magnification in adjacent lower panels. Bar, 500 μm. *F*, bone mineral density (BMD) of femurs from 15-week-old indicated mice, as measured by DEXA. Data represent mean BMD (mg/cm2) of each time point ± SD (n = 6, ∗*p* < 0.05).

Phenotypes of ttw mice fed a HPD are reportedly rescued by a high phosphate/low vitamin D diet (HPLD) (20). Thus, we fed 8-week-old Col2 Cre/Enpp1 cKO mice either a HPD or HPLD. The significant body weight loss seen in Col2 Cre/Enpp1 cKO mice fed a HPD was significantly rescued by feeding the HPLD (Fig. 6, *A* and *B*). At 15 weeks of age, when mice had been fed the HPD or HPLD for 7 weeks, micro CT imaging showed that ectopic ossification around the intervertebral disc seen in Col2 Cre/Enpp1 cKO mice was enhanced by the HPD but not by the HPLD (Fig. 6*C*).

Von Kossa staining of cervical spine confirmed enhancement of ectopic calcification in spine by the HPD but not the HPLD in Col2 Cre/Enpp1 cKO mice (Fig. 6*D*). Similarly, histological analysis using Alizarin red staining showed that ectopic calcification in kidney was accelerated by a HPD but not a HPLD in Col2 Cre/Enpp1 cKO mice (Fig. 6*E*). Furthermore, bone mineral density was significantly decreased in Col2 Cre/Enpp1 cKO mice fed a HPD relative to what other three groups, but this decrease was not seen in Col2 Cre/Enpp1 cKO mice fed the HPLD (Fig. 6*F*). These results indicate that elevated vitamin D3 levels may underlie induction of aging phenotypes of Col2 Cre/Enpp1 cKO mice fed an HPD.

## Discussion

Aging is a multifactorial process, and its rate is thought to be determined by a combination of individual and environmental factors (29, 30, 31). However, it has been unclear whether a particular organ or tissue controls aging. Here, we show that chondrocyte-specific Enpp1 deletion results in a significantly shortened life span. Thus, cartilage tissue plays a role in controlling systemic aging and that Enpp1 activity functions in this process.

Among factors that control aging, genetic factors are known to cause premature aging in a variety of disorders (29, 32), such as Werner syndrome or Hutchinson-Gilford-Progeria syndrome (33, 34). However, phenotypes associated with these diseases are apparent very early after birth in humans and represent pathological rather than general aging (35). Here, we report that Col2 Cre/Enpp1 cKO mice fed a HPD as adults show various phenotypes characteristic of normal aging, such as ectopic calcification of kidney and osteoporosis. In mice, the Col2a1 promoter is highly active in early development and then becomes less so in adult animals (26, 27). Since Enpp1 is expressed in growth plate chondrocytes in developing stages (Fig. 1*D*), we chose to use Col2a1 Cre mice to delete the Enpp1 gene in chondrocytes. Meanwhile, Aggrecan Cre mice have been used by others to examine postnatal phenotypes such as osteoarthritis (36). Nonetheless, since Col2 Cre/Enpp1 cKO mice exhibit significantly shortened life span compared with controls, Enpp1 expression in chondrocytes likely plays a role in regulating systemic aging throughout life.

Proper regulation of phosphate metabolism is reportedly crucial to suppress ectopic calcification, such as that occurring in dialysis patients when renal function is impaired (1, 37). In this study, we found that cartilage tissue regulates systemic ectopic calcification, a finding that was unanticipated. Articular cartilage maintains homeostasis by not calcifying, which also contributes to prevention of global calcification. It is also well known that PPi, which is produced prior to phosphorus synthesis, is the most important factor in preventing calcification (10). Enpp1 activity regulates PPi levels, and Enpp1-deficient ttw mice exhibit reduced blood PPi levels (Fig. 2*E*). Here, we show that Col2 Cre/Enpp1 cKO mice also exhibit significantly reduced blood PPi phosphate levels, and thus cartilage tissue is a likely regulator of systemic PPi levels, ectopic calcification, and aging through Enpp1 activity. However, phenotypes seen in Col2a1 Cre/Enpp1 cKO mice were not completely identical to those seen in CAG Cre/Enpp1 cKO mice, and serum PPi levels were significantly lower in CAG Cre/Enpp1 cKO compared to Col2a1 Cre/Enpp1 cKO mice (Figs. 2*D* and 3*E*). These observations suggest that Enpp1 expressed below the detection limit of bioluminescence or fluorescence immunostaining in noncartilaginous tissues has activity similar to Enpp1 expressed in cartilaginous tissues.

Osteoarthritis due to cartilage degeneration is a typical disease of aging, but conversely, degeneration may be caused by cartilage tissue dysfunction. In fact, ectopic ossification such as osteophytes is observed in osteoarthritis, and osteoarthritis reportedly correlates with osteoporosis, another age-related disease (38, 39). We demonstrate that Col2 Cre/Enpp1 cKO mice fed a HPD exhibit significantly reduced bone mass. These findings suggest that ectopic calcification decreases proper calcium deposition in bones, leading to osteoporosis due to reduced bone mineral density. Indeed, calcification of the abdominal aorta is known to correlate with osteoporosis development (40, 41). Maintaining homeostasis of cartilage tissue may be required to control systemic aging, including osteoporosis.

Currently, calorie restriction is the only known evidence-based method across species that slows the aging process (42, 43). By contrast, high caloric intake leads to several diseases that shorten life span, such as atherosclerosis (42, 43). We show that conditions observed with high caloric intake, including ectopic calcification in kidney and osteoporosis, are also seen in Col2 Cre/Enpp1 cKO mice fed an HPD. Regulation of phosphorus metabolism by Enpp1 may have similar significance to calorie restriction in aging regulation.

Low blood vitamin D levels are a well-known risk factor for osteoporosis development and fragility fractures in the elderly (41, 44). However, this study showed that vitamin D is a risk factor for ectopic calcification and osteoporosis when Enpp1 function is disrupted. Control of phosphorus metabolism and vitamin D levels may be important for future control of aging and prevention of osteoporosis fragility fractures. Taken together, our study indicates that cartilage represents as a control center of systemic aging *via* Enpp1.

## Experimental procedures

### Mice and diets

The ttw mice, a spontaneous mutant harboring a mutation in the *Enpp1* gene (16, 17), were maintained as described (20). The Enpp1-EGFP-luciferase reporter mice (Accession No. CDB0010E: https://large.riken.jp/distribution/mutant-list.html) were established by knocking in a chimeric sequence of EGFP and firefly luciferase at the start codon of exon 1 of mouse *Enpp1* gene using CRISPR/Cas9-mediated genome editing in zygotes as previously described (45). Enpp1 flox/flox mice were generated by creating loxP sequences at both ends of *Enpp1* exon 18, since ttw mice carry a nonsense mutation in that exon (17). CAG Cre mice were prepared as described (46), as were Col2 Cre mice (47). WT mice were obtained from CLEA Japan, Inc (Meguro). Primers for genotyping PCR were as follows.

Enpp1-EGFP-luciferase reporter-forward:5′-CGACCTACCAGCGACAGC-3′

Enpp1-EGFP-luciferase reporter-reverse:5′-TCATCGACAAGTACGACCTAAGCA-3′

Enpp1-flox/flox-forward:5′- CACATCTCTCTGTGTGTGTGCA-3′

Enpp1-flox/flox-reverse:5′- GCAGTAAGTTGGGGGTTGGGCC-3′

Mice were fed either a normal phosphate diet (1% phosphate, ND), a HPD (1.5–2% phosphate), or a HPLD (1.5% phosphate) starting at 8 weeks of age for at least 2 weeks or for indicated periods. The HPLD contains 0 units/100 g Vitamin D units. The other diets contain Vitamin D 240 units/100 g. Animal experiments were approved by the Institutional Animal Ethics Committees of Kumamoto University (approval A2020-127, A 2022-024) and the Institutional Animal Care and Use Committee of RIKEN Kobe Branch (Approval number: A2001-03).

### Bioluminescence imaging

The IVIS spectrum cooled charge-coupled device optical macroscopic imaging system (Caliper Life Sciences) was used for *in vitro* and *in vivo* bioluminescence imaging, as reported by others (48). *In vivo* imaging was performed 15 min after i.p. injection of D-luciferin (0.15 mg/g body weight) (Cayman Chemical Company) with the field-of-view set at 13.2 cm, since photon count was most stable during this period. Intensity peaked between 15 and 30 min after i.p. D-luciferin injection. Mice were euthanized 20 min after injection. Each tissue was then isolated, followed by *ex vivo* bioluminescence imaging. Integration time was fixed at 15 s for each image.

For *in vitro* imaging, chondrocytes were pellet cultured and treated with D-luciferin (0.5 mM), and images were taken 15 min later. Integration time was fixed at 60 s for each image.

### Quantitative PCR analysis

Total RNAs were isolated from indicated tissues using TRIzol reagent (Invitrogen Corp). RNA samples were quantified based on A260/A280 ratios using a Thermo Scientific NanoDrop One spectrophotometer (Thermo Fisher Scientific). Samples with A260/A280 ratios >1.8 were considered pure and subjected to cDNA synthesis. A Prime Script RT reagent Kit (Takara Bio Inc) was used for reverse transcription of mRNA. cDNA was synthesized using a Thermal Cycler Dice (Takara Bio Inc) according to the manufacturer’s instructions.

Quantitative real-time PCR was performed using TB Green Premix ExTaq II reagent (Takara Bio Inc) and a Thermal Cycler Dice (Takara Bio Inc) according to the manufacturer’s instructions. β-actin (Actb) expression served as an internal control. Primers for real-time PCR were as follows.

*mEnpp1*-forward: 5′-AAGCATGGTGCTGAAGTTGACTC-3′

*mEnpp1*-reverse: 5′-TGGGATGACTTGGGTTGTAAATG-3′

*Klotho*-forward: 5′-GACAATGGCTTTCCTCCTTTACCT-3′

*Klotho*-reverse: 5′-TGCACATCCCACAGATAGACATTC-3′

*Cyp27b1*-forward: 5′-ACTCAGCTTCCTGGCTGAACTCTT-3′

*Cyp27b1*-reverse: 5′-GTAAACTGTGCGAAGTGTCCCAAA-3′

*β-actin (Actb)*-forward: 5′ – AAGTGTGACGTTGACATCCG-3′

*β-actin (Actb)*-reverse: 5′ – GATCCACATCTGCTGGAAGG -3′

### micro CT

Vertebral bones and surrounding tissues, including intervertebral discs, the posterior longitudinal ligament, and Achilles tendon, were scanned by a microcomputed tomography (SkyScan1176, Bruker). Two-dimensional regions of interest were created at the level of the cervical spine and Achilles tendon.

### Histopathological analysis

Tissue samples were fixed 2 h in 4% PFA/PBS at 4 °C, treated with 20% sucrose in PBS, and frozen in SCEM-L1 compound (Section-lab). Samples were then cryosectioned to a thickness of 4 μm and stained with hematoxylin and eosin and von Kossa or Alizarin Red. Sections were observed under a microscope (BZ-X700 microscope, Keyence), and digital images were captured.

### Immunohistochemistry

Antigen retrieval was performed using 0.05% proteinase K (Kanto chemical co, INC) in PBS for 15 min at room temperature. Sections were then incubated in 3% BSA in PBS for blocking and stained with primary antibodies at 4 °C. Subsequently sections were washed and incubated with Alexa488-conjugated goat anti rabbit Ig’s antibody (diluted 1:400), Alexa488-conjugated donkey anti goat Ig’s antibody(diluted 1:200) or Alexa594-conjugated Donkey anti rabbit Ig’s antibody (diluted 1:200) (Life Technologies Corporation) for 120 min at 4 °C. Slides were mounted and counterstained with Vectashield mounting medium for fluorescence with DAPI (Vector Laboratories) and were imaged under a fluorescence microscope (BZ-X700). Primary antibodies used were anti-GFP (diluted 1:1000; Code No. 598, MBL), Goat anti-EGFP (diluted 1:250; ab6673, Abcam), rabbit anti-Type 2 collagen (diluted 1:200; ab34712, Abcam), and rabbit anti-CDKN2A/p16INK4a (diluted 1:200; SAB5700620, Sigma-Aldrich).

### Chondrocyte culture

Chondrocytes from rib cartilage were prepared from 1-week-old C57/B6 mice and from Enpp1-luciferase-EGFP KI mice, as previously reported (49).

### Measurement of plasma PPi

For PPi analysis, plasma was collected from mice, and samples were filtered through a 30 kDa membrane (PALL) by centrifugation to remove platelets. Plasma was frozen at −80 °C within 1 h of blood collection for single use. Measurement of plasma PPi was performed using ATP sulfurylase with slight modifications of previous protocols (50, 51, 52).

### Flow cytometry

Bone marrow was extracted from mouse femur and tibia. Using a 70 μm cell strainer (pluriSelect), suspensions were filtered to remove debris and then layered on lympholyte-M medium (Cedarlane Laboratories Ltd) for purification. Cells were then stained 20 min with an appropriate monoclonal antibody. Antibodies used were anti-mouse CD31-PE (102508, BioLegend), anti-mouse CD45-FITC(11-0451-82, Thermo Fisher Scientific), and anti-mouse PDGFRa-APC (17-1401-81, Thermo Fisher Scientific). Flow cytometry was performed with FACSVerse (BD Biosciences), and the data were analyzed using Flowjo software (BD Biosciences). Gates for MSCs were defined as positivity for PDGFR-α and negativity for CD31, CD45, according to the fluorescence intensity of the isotype control.

### Analysis of skeletal morphology

Bone mineral density of whole femurs was measured using Dual-energy X-ray absorptiometry.

### Statistical analyses

All results are reported as the mean ± standard error (SD). We used the Mann–Whitney U test and Kruskal–Wallis test to calculate *p* values. A *p* value < 0.05 was considered statistically significant (∗*p* < 0.05; ∗∗*p* < 0.01; ∗∗∗*p* < 0.001).

## Data availability

A summary of all original data are presented in the manuscript, including all individual data points collected. Unprocessed data are available on request from the corresponding author: Takeshi Miyamoto (miyamoto.takeshi@kuh.kumamoto-u.ac.jp).

## Supporting information

This article contains supporting information.

## Conflict of interest

The authors declare no conflict of interest with the contents of this article.
