# Supplementary material for: Cartilage tissues regulate systemic aging via ectonucleotide pyrophosphatase/phosphodiesterase 1 in mice
Source: J Biol Chem. 2023 Nov 30;300(1):105512. doi: 10.1016/j.jbc.2023.105512 (PMC10777000; doi:10.1016/j.jbc.2023.105512)
Supplement: Supporting Figures S1–S7 [file mmc1.pdf]

Supplementary Figure 1

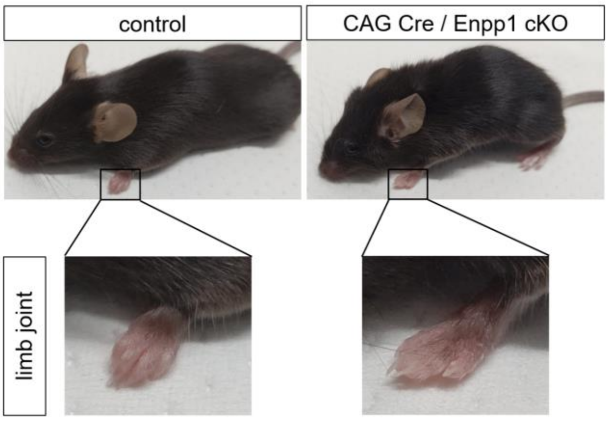

Supplementary Figure 1. Appearance of CAG Cre/Enpp1 cKO mice.

Gross appearance (upper) and forelimbs (lower) of representative 8-week-old *Enpp1*$^{flox/flox}$ (control) and CAG Cre/Enpp1 cKO mice.

Supplementary Figure 2

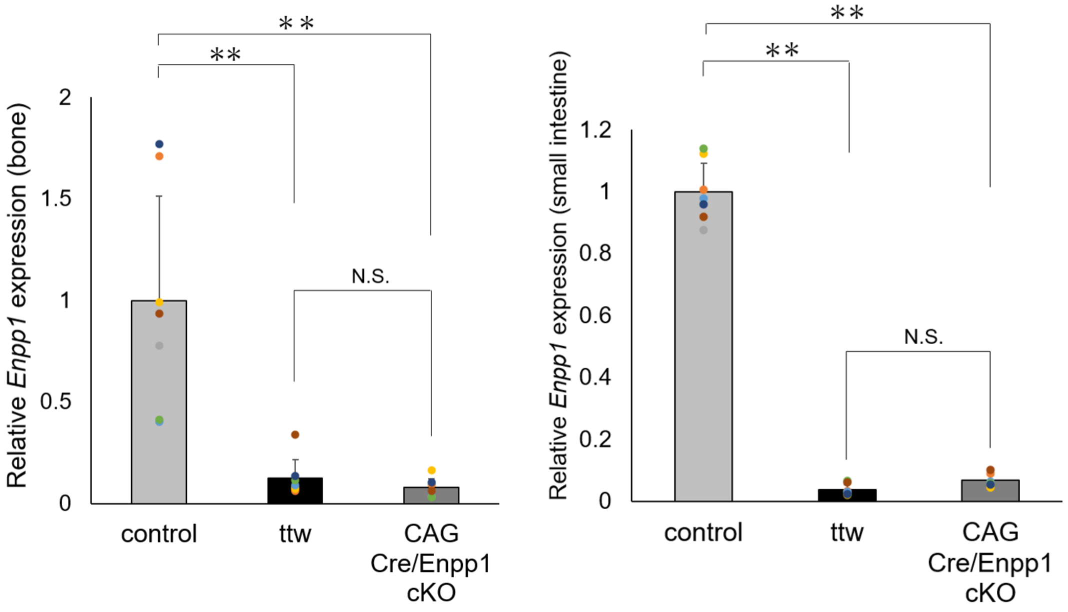

Supplementary Figure 2. CAG Cre/Enpp1 cKO mice show significantly decreased *Enpp1* expression in bone and small intestine.

RNA was extracted from femoral bone and small intestine of 8-week-old *Enpp1*$^{flox/flox}$ (control), ttw, or CAG Cre/Enpp1 cKO mice, and *Enpp1* expression was analyzed by realtime PCR. Data represents mean *Enpp1* expression relative to *β-actin* (n = 7, **p < 0.01. ns, not significant).

Supplementary Figure 3

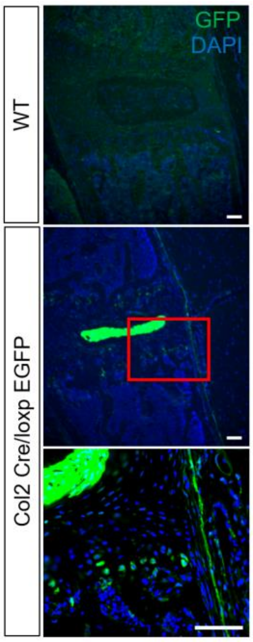

Supplementary Figure 3. EGFP expression is detected in chondrogenic cells in Col2 Cre/loxP EGFP reporter mice.

Undecalcified frozen sections of cervical spine from 4-week-old wild-type (WT) and Col2 Cre/loxP EGFP reporter mice were stained with rabbit anti-EGFP antibody (diluted 1:1000) followed by Alexa488-conjugated goat anti-rabbit Igs antibody (diluted 1:400), and observed under a fluorescence microscope. Nuclei are DAPI-stained. Bar, 50μm.

Supplementary Figure 4

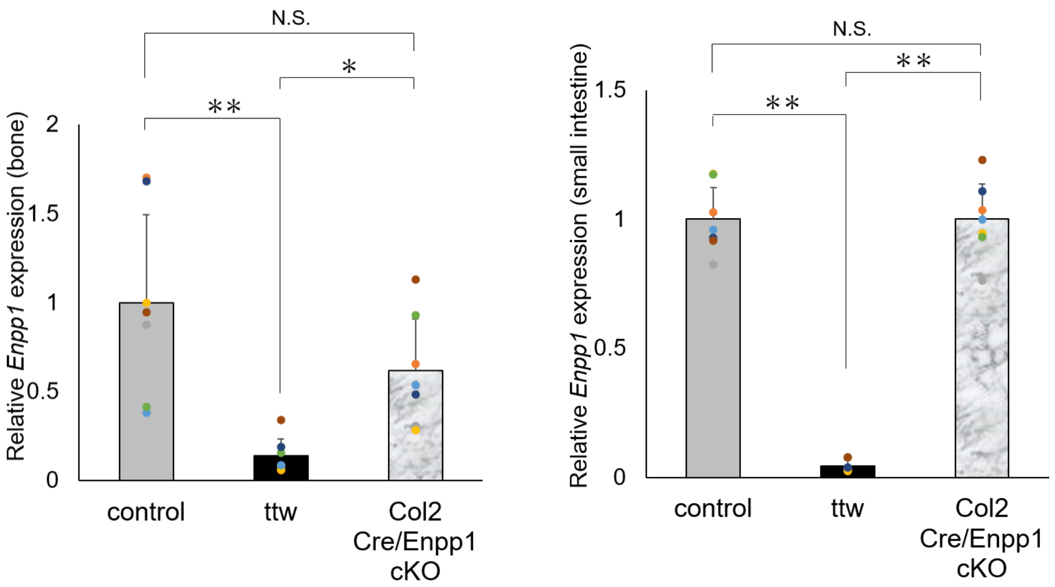

Supplementary Figure 4. *Enpp1* is expressed in bone and small intestine of Col2 Cre/Enpp1 cKO mice.

RNA was extracted from femoral bone and small intestine of 8-week-old *Enpp1*$^{flox/flox}$ (control), ttw, and Col2 Cre/Enpp1 cKO mice, and *Enpp1* expression was analyzed by realtime PCR. Data represents mean *Enpp1* expression relative to *β-actin* ($n = 7$, $*p < 0.05$, $**p < 0.01$. ns, not significant).

Supplementary Figure 5

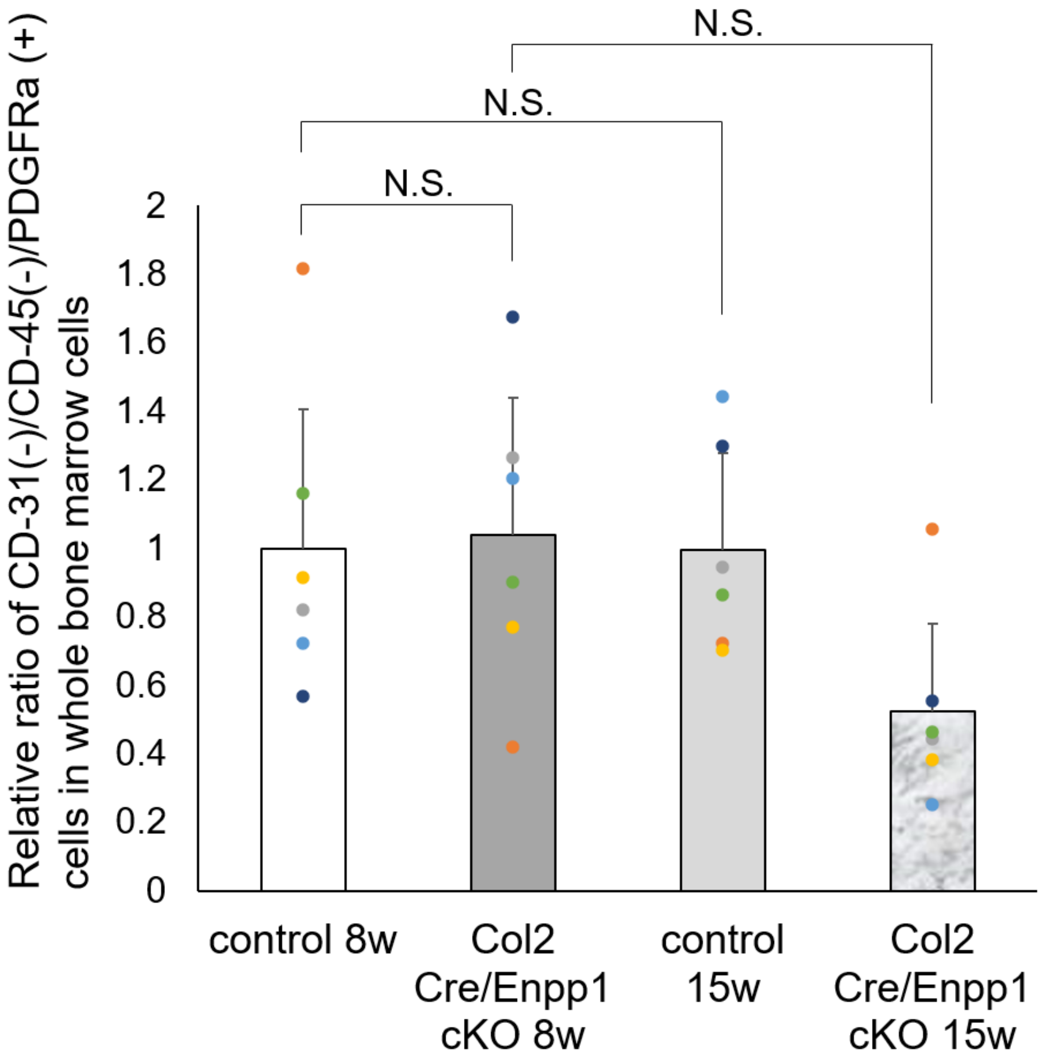

Supplementary Figure 5. The frequency of mesenchymal stem/progenitor cells in bone marrow decreased with age in Col2 Cre/Enpp1 cKO mice.

Bone marrow was extracted from mouse femur and tibia of control and Col2 Cre/Enpp1 cKO mice at both 8- and 15-weeks of age. Cells were stained with anti-mouse CD31-PE, anti-mouse CD45-FITC and anti-mouse PDGFRa-APC, and analyzed by flow cytometry. The graph shows the average percentages of CD-31(-)/CD-45(-)/PDGFRa (+) cells among 10^6 input cells (n = 6). Data represents mean ratio of CD-31(-)/CD-45(-)/PDGFRa (+) cells in whole bone marrow cells in indicated mice relative to that in 8-week-old control mice (n = 6, ns, not significant).

Supplementary Figure 6

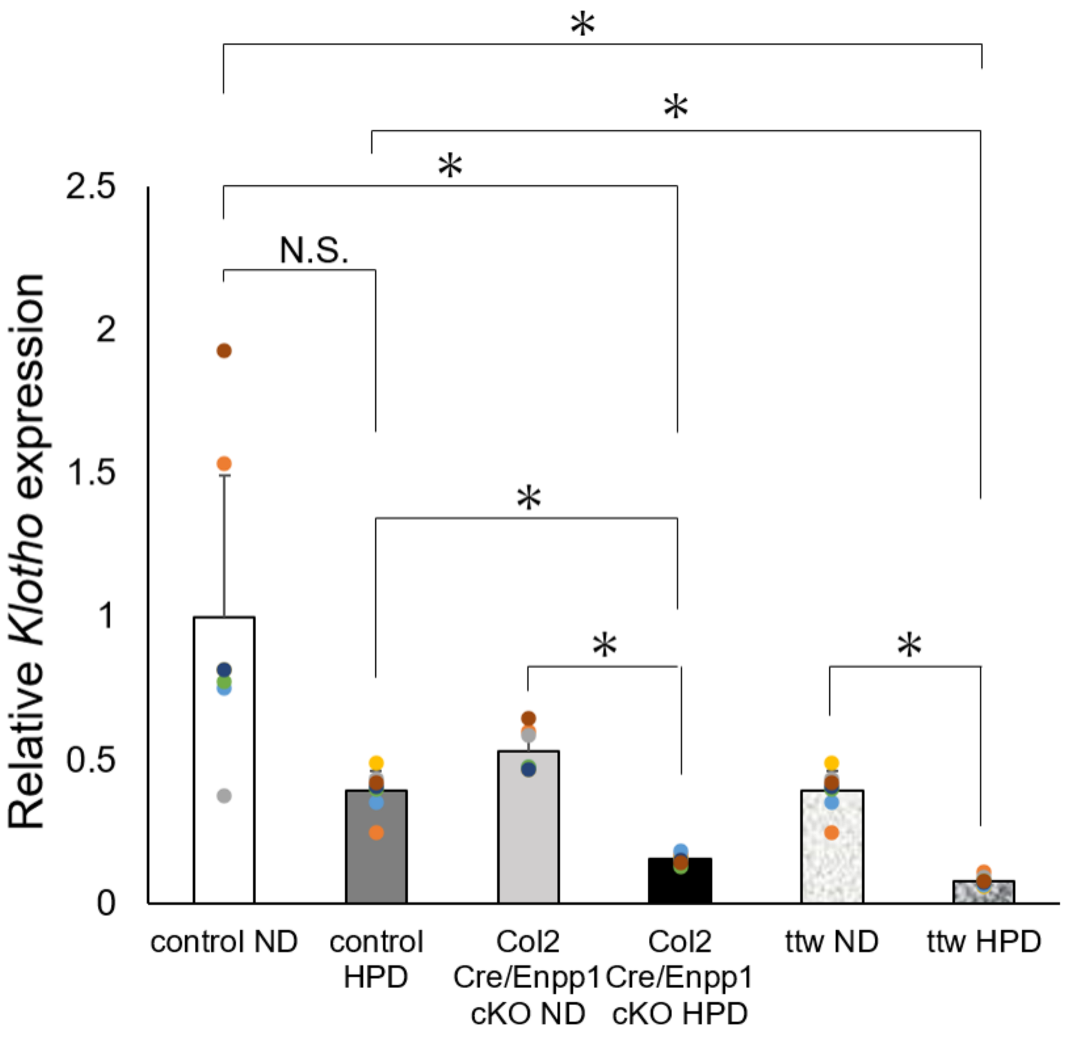

Supplementary Figure 6. Klotho expression in kidneys decreases by feeding a high phosphate diet in either of mice.

Eight-week-old *Enpp1*$^{flox/flox}$ (control) and Col2 Cre/Enpp1 cKO mice were fed a normal diet (ND) or high phosphate diet (HPD) for 7 weeks. Also, 8-week-old *Enpp1*$^{ttw/ttw}$ mice were fed a ND or HPD for 2 weeks. RNA was then extracted from kidney tissue in each group and *Klotho* expression was analyzed by realtime PCR. Data represents mean *Klotho* expression relative to *β-actin* ± S.D. (n = 7, *p < 0.05. ns, not significant).

Supplementary Figure 7

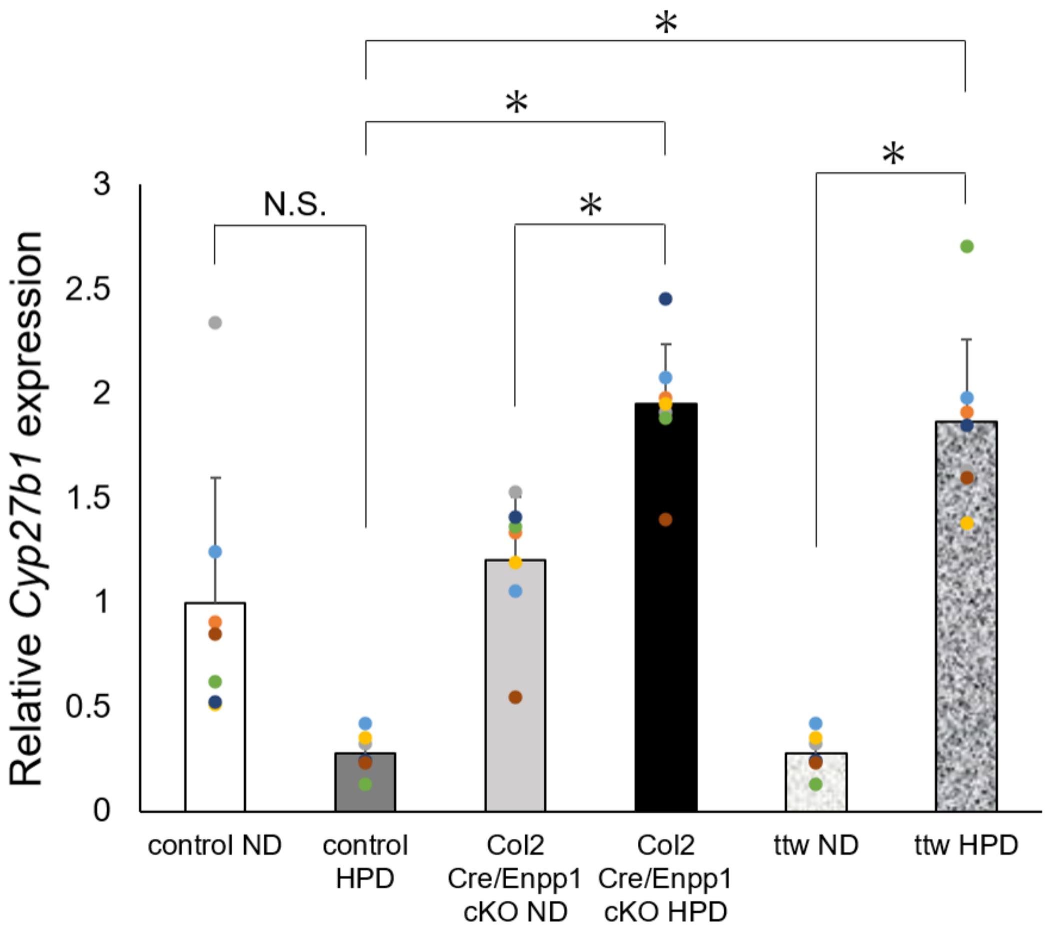

Supplementary Figure 7. *Cyp27b1* expression in kidney is elevated by feeding a high fat diet in Col2 Cre/Enpp1 cKO and *Enpp1*$^{ttw/ttw}$ mice.

Eight-week-old *Enpp1*$^{flox/flox}$ (control) and Col2 Cre/Enpp1 cKO mice were fed a normal diet (ND) or high phosphate diet (HPD) for 7 weeks. And, 8-week-old *Enpp1*$^{ttw/ttw}$ mice were fed a ND or HPD for 2 weeks. RNA was then extracted from kidney tissue in each group and *Cyp27b1* expression was analyzed by realtime PCR. Data represents mean *Cyp27b1* expression relative to *β-actin* ± S.D.(n = 7, *p < 0.05. ns, not significant).
